# Diagnosis and Treatment of Ocular Pain: the Ophthalmologist’s Perspective

**DOI:** 10.1007/s40135-017-0152-1

**Published:** 2017-11-02

**Authors:** Deborah S. Jacobs

**Affiliations:** 1 BostonSight, Needham, MA USA; 2 Harvard Medical School, Boston, MA USA; 3 Mass Eye & Ear Cornea Service, Boston, MA USA

**Keywords:** Ocular pain, Neuropathic pain, Dry eye, Diagnosis, Treatment

## Abstract

**Purpose of Review:**

The aim of this review is to help ophthalmologists and other clinicians understand and treat ocular neuropathic pain.

**Recent Findings:**

Advances in the field of neurophysiology of ocular sensations explain why some cases of dry eye may represent a pain syndrome rather than a problem related to tear status. Principles related to management of pain syndromes such as persistent post-operative pain and complex regional pain syndrome are relevant to the care of these patients.

**Summary:**

Specific strategies for the ophthalmologist, including multimodal therapy comprised of local and systemic approaches, can be helpful in the care of patients with problematic ocular pain. Rather than dismiss these syndromes and these patients, ophthalmologists can serve these patients and the profession well by incorporating an understanding of ocular neuropathic pain into their practice and by collaboration in the care of patients with problematic ocular pain.

## Introduction

People with ocular pain typically present or are referred to ophthalmologists or optometrists for evaluation. When there is recent trauma or surgery, or signs of an infectious or inflammatory process, treatment of the underlying process or pathologic abnormality usually results in resolution of pain. These situations represent physiologic or nociceptive pain. Such pain associated with surgery, injury, infection, or inflammation at the front of the eye is typically treated with topical steroid, topical NSAID, systemic NSAID, lubricant ointment, gel or drops, bandage contact lens, or a few doses of oral opiate or topical anesthetic. When the patient complains of symptoms out of proportion to clinical signs or with no apparent previous insult, the presentation enters the realm of pathologic or neuropathic pain, which is the topic to be addressed here. Furthermore, this review will address only pain that maps to the ocular surface or cornea. Headache, orbital, retrobulbar, and facial pain are beyond the scope of this review.

## Neurophysiology of Ocular Sensation

The last decade has brought advances in the understanding of the neurophysiology of sensation from the ocular surface, largely from the study of anatomy, physiology, and neuropharmacology of mammalian sensory, motor, and autonomic pathways. Nociceptors at the ocular surface respond to mechanical, chemical, and thermal stimuli. The neurologic pathways they feed into serve as defense mechanisms for the transparent cornea against an adverse environment. In particular, there have been advances in understanding regarding nociceptors that detect cooling. These corneal nerves function as evaporation detectors and serve to maintain a moist ocular surface through autonomic connections to the blink apparatus and to the lacrimal, mucus, and lipid secreting glands of the ocular surface [1••].

## Dry Eye as a Pain Syndrome

Dry eye, dry eye disease, and dry eye syndrome are names for a common clinical presentation that has received increasing attention over the last two decades. There remains great unmet need in the treatment of dry eye. In the decade since the first International Dry Eye Workshop [2], there has been a proliferation of postulated mechanisms and treatments, such that the length of report of from the second International Dry Eye Workshop is several times that of the first [3]. What has emerged is the understanding that some patients classified as having dry eye may indeed have problem not related to moisture on the eye, per se, but rather a perturbance of pain and sensation [4]. Patients with derangement of this system may report symptoms of dry eye, that is burning, grittiness, irritation, photophobia, and tired eyes that want to close, but have little in the way of signs of ocular surface breakdown. Some fraction of dry eye patients who have symptoms out of proportion to signs may be more properly classified as having ocular pain, which might further explain the lack of effectiveness of treatment directed toward the tear film itself. These patients may be dismissed as hysterical or malingering, by clinicians who do not have an appreciation of pain syndromes as they might affect the eye [5–7].

Clinicians have some appreciation of neuropathic pain that can involve the eye in the example of post-herpetic neuralgia (PHN). Approaches to prevention and treatment are accepted and undertaken in the mainstream, typically involving early treatment with antivirals as a prevention again PHN. During the eruption, pain can be treated with gabapentin, TCAs, and other anti-epileptics sometimes used for pain. In patients with history of dermatomal eruption and parathesia, pain in the same distribution is rarely dismissed. Principles related to an understanding of this syndrome may be useful in treating other types of neuropathic eye pain.

## Diagnosis

Neuropathic ocular pain is a challenging frontier because there are no agreed upon diagnostic criteria. Indeed, even within the field of pain medicine, the taxonomy of pain syndromes and the definition of neuropathic pain continue to evolve [8]. Current definition of neuropathic pain required evidence of damage or injury to the somatosensory nervous system, and diagnosis is stratified as possible, probable, or definite neuropathic pain.

The diagnosis of neuropathic eye pain remains clinical, with testing serving to support the diagnosis. Principals from pain medicine can be applied, with patients using pain scales to quantify their symptoms. Certain descriptors may be suggestive of neuropathic etiology, such as “burning,” but none is specifically diagnostic.

### Questionnaires and Surveys

Validated survey instruments for eye disease such as the OSDI [9] and DEQ [10] can be useful for quantifying symptoms or quality of life, categorizing patients, and monitoring response to intervention. Some are quite easy to use and score, yet generally they are used only as research tools. The OPAS is a recently validated survey specifically for ocular pain [11].

### Sensory Testing

Testing can be supportive, but again there are no standard criteria. Testing can help to clarify if the pain syndrome remains peripherally mediated or has become centralized. Testing of structure and testing of function are two areas currently being studied. Elimination of symptoms with an in-office trial dose of topical anesthetic suggests that pain is peripherally mediated by the peripheral nociceptors. Persistence of symptoms after administration of topical anesthetic suggests that pain is mediated centrally [12, 13].

Local testing of sensory thresholds or sensitivity may also point to neuropathic pain, for example if local nociception is reduced in the presence of other pain symptoms. The Cochet-Bonnet aethesiometer can detect reduced mechanosensitivity, and the Belmote gas aethesiometer is useful in characterized sensitivity thresholds. Study of sensory pain processing can point to neuropathic etiology of dry eye symptoms. At this time, there is no ocular sensory testing that is diagnostic for any particular ocular pain syndrome.

Allodynia and hyperalgesia are common features of centralized neuropathic pain [14]. There are no standard ocular scales for these, but certainly, their presence can be documented as part of a clinical exam. Allodynia at the ocular surface is typically reported as sensitivity to moving air, such as a dashboard blower fan, and hyperalgesia as disproportionate pain from a mildly noxious stimulus such as a medicated or lubricant drop or the placement of a Schirmer test paper strip.

Testing of non-ocular sensory thresholds or sensitivity may reveal the presence of systemic neuropathy or suggest central sensitization that might be the basis for ocular pain [13, 15]. Identification and treatment of such neuropathy might suggest a pathway for management of ocular symptoms. The presence of altered thresholds might suggest that additional measures be undertaken to prevent centralization of pain in individuals who are susceptible.

### Imaging

#### Confocal Microscopy

Advances in corneal confocal microscopy have allowed for the recent study of the corneal sensory sub-basal nerve plexus and of corneal immune cells. Reduced nerve density, increased tortuosity, tangles, and presence of dendritic cells may be markers of injury, physiologic recovery, or pathologic recovery. There are discrepancies in reports of nerve morphology in dry eye disease and after trauma, suggesting the sensory plexus may have dynamic features that are incompletely understood [4]. Advances in image analysis may allow for standard criteria for diagnosis and as a metric for treatment of physiologic and pathologic pain mapping to the ocular surface. A recent report is provocative in that it found only eyes of patient with dry eye disease and normal nerve density responded to local treatment with topical lubricant or steroid, [16] suggesting that any change in morphology is associated with changes in central processing that may require central or systemic approaches.

#### fMRI

Functional imaging can detect photophobia and pain related to insult at the ocular surface [17] and on that basis holds promise for diagnosis of physiologic vs pathologic ocular pain. As yet there are no standard approaches for evaluating patients with eye pain.

## Treatment

### Approaches and Goal

Approaches to the treatment of ocular pain come from the fields of neuroscience, neurology, and pain management rather than the field of ophthalmology. Clinicians should be alert and attuned to the possibility that a patient might have a pain syndrome rather than a report of physiologic nociception. Symptoms are, of course, the manifestation of signaling and should be the focus of treatment. Continued signaling converts peripheral to centralized pain and acute to chronic pain. The goal of treatment is to reduce signaling. This might seem obvious, since of course the patient is seeking treatment for symptoms, but when it is recognized that the syndrome is pathologic pain, rather underlying organ or tissue disease one is more likely to pick therapies geared directly to reducing signaling.

When there is prior injury or surgery, such as LASIK or recurrent erosion or radiation, one might consider this a parallel to persistent post-operative pain syndrome or complex regional pain syndrome [18•, 19••]. When pain projected to the ocular surface manifests in a patient with history of pain elsewhere, one might consider approaches as for systemic neuropathy or neuralgia.

### Local Treatment for Peripheral Signaling

If a peripheral process is suspected, that is pain can be reduced or eliminated with topical anesthetic, it may be worthwhile to attempt to reduce any pathological signaling of nociceptor and promote physiologic healing of damaged nerves. Local therapies that might reduce local inflammation such as topical steroid, topical immunomodulators such as cyclosporine or lifitgrast, autologous serum tears, and NSAIDS are worth a therapeutic trial. Since the nerves are superficial and dose-related response has never been shown, it is judicious to use “soft steroid” such as lotemax, FML, in tapering pulse therapy or at very low suppressive dosing to reduce likelihood of side effects. Topical NSAIDS are generally used to manage post-PRK pain. Additionally, diclofenac has been found effective in reducing pain signaling in a small series of normal controls [20]. NSAIDs may be helpful in reducing peripheral signaling, but benefit must be weighed against known risk of sterile ulceration with chronic use of any NSAID, particular in patients with tendency for surface breakdown, reduce corneal sensation, or underlying autoimmune disease. Patients with “burning” or other symptoms of evaporation hypersensitivity may respond to punctal occlusion, frequent or viscous lubricants, goggles, and wraparound glasses and to the use of soft contact lens, scleral lens, or PROSE treatment as shields to evaporation.

### Systemic Treatment for Peripheral Signaling or for Centralized Pain

In patients with longstanding symptoms of more than several months, or if symptoms are not eliminated with topical anesthetic, then it is likely there is some central mediation of pain. Gabapentin and pregabalin have been shown to reducing post-operative pain after PRK [21, 22] and after eyelid surgery [23] and they are labeled for PHN. One might conclude that together, there is support for the use of these systemic agents more broadly in pain syndromes involving the eye. Furthermore, among anti-epileptics used for chronic pain, clinical trial evidence supported the use of only gabapentin and pregabalin in some neuropathic pain conditions (painful diabetic neuropathy, post-herpetic neuralgia, and central neuropathic pain) and fibromyalgia [24••]. Evidence supporting the use of tricyclic antidepressants is poor although they are frequently used and may be helpful in some patients. When eye pain is part of a generalized pain syndrome such as fibromyalgia, pregabalin and/or duloxetine, both labeled for fibromyalgia may be helpful [25, 26]. There is some evidence that combination therapy rather than monotherapy has a role in chronic neuropathic pain [27]. Strategies for selection of systemic agents for non-ocular neuropathic pain by authors reviewing ocular neuropathic pain is consistent with the summary presented here [28].

### Stimulation Therapies

Stimulation therapies, peripheral and central, invasive and non-invasive, including acupuncture, DBS, tDCS, rTMS, and Calmare scrambler, are used in pain management. The quality of evidence supporting their use remains only poor to moderate [29] and none have yet to be validated for ocular neuropathic pain.

### Psychological Approaches

Cognitive-behavior therapy may be helpful in patients with chronic pain syndromes [30]. There are no reports specifically on psychologic treatment of dry eye, corneal pain, or ocular surface pain. Patient with corneal projected pain may resistant to the idea that psychological approaches will be helpful.

## Clinical Perspectives

Pain management is field with no simple solutions. A review of reviews(!) of anti-epileptics for neuropathic pain concludes that it may be worthwhile to seek evidence about strategies rather than interventions to produce overall best results in a population, in the shortest time, at the lowest cost to health providers [24]. With that in mind, the following strategies are suggested.

### Strategy 1—Name This as a Nerve Problem

When evaluating and treating patients with neuropathic pain, it is helpful to describe the problem as a nerve problem rather than eye problem. Patients generally understand and appreciate this distinction. Because eye pain, as with the pain mediated via the trigeminal nerve such as toothache and classic trigeminal neuralgia, is perceived acutely, patient may benefit from reassurance that no blinding process is occurring and that their nerves are sending “a false alarm.”

### Strategy 2—Recognize that a Single Cure Is Unlikely

Given that the pain is not nociceptive/pathologic, there is rarely a silver bullet or single treatment that eliminates pain entirely. It is important for both clinicians and patients to realize this, and to address the expectation that the next therapeutic option is going to solve the problem. Patients seem to benefit from multimodal treatment that includes local, systemic, and cognitive-behavioral approaches.

### Strategy 3—Arrange for Frequent Scheduled Visits

Pain makes patients anxious and anxious patients are more susceptible to pain. Furthermore, catastrophization is not uncommon in patients with pain syndromes. Rather than a specific intervention, frequent scheduled visits, rather than discharge instructions “return in 1 year, or as needed,” may avoid urgent calls for episodic evaluation. Prospective scheduling at 4–8-week intervals may eliminate urgent episodic visits and allow for assessment and reassurance. The interval between visits can be increased as the acuity of the syndrome decreases.

### Strategy 4—Collaborate with Other Clinicians

Collaborative care with primary care doctor, neurologist, physiatrist, rheumatologist, or psychiatrist may be warranted, particularly when there is centralized pain and systemic agents are required. These clinicians may not be comfortable assessing ocular “endpoints” of treatment, and for that reason, continued ongoing ophthalmologic care is helpful. That is, the patient should not be dismissed to a neurologist, for example, but followed along jointly. New understanding of the mechanisms of neuropathic pain, new diagnostic procedures, and personalized intervention highlights the necessity of an individualized multidisciplinary approach to the management of neuropathic pain [31].

## Summary

Ophthalmologists appreciate the instantaneous gratification that arises when restoring vision with cataract surgery or LASIK. It is no less gratifying to help a patient with disabling neuropathic pain return to “normal” life. Rather than dismiss these syndromes and these patients, ophthalmologists can serve these patients and the profession well by incorporating an understanding of ocular neuropathic pain into their practice and by collaboration in the care of patients with problematic ocular pain.
